# Normal Weight Estonian Prepubertal Boys Show a More Cardiovascular-Risk-Associated Adipose Tissue Distribution than Austrian Counterparts

**DOI:** 10.1155/2013/506751

**Published:** 2013-01-14

**Authors:** Sandra J. Wallner-Liebmann, Reinhard Moeller, Renate Horejsi, Toivo Jürimäe, Jaak Jürimäe, Jarek Mäestu, Priit Purge, Meeli Saar, Erwin Tafeit, Petra Kaimbacher, Renate Kruschitz, Daniel Weghuber, Wolfgang J. Schnedl, Harald Mangge

**Affiliations:** ^1^Center of Molecular Medicine, Institute of Pathophysiology and Immunology, Medical University of Graz, Heinrichstraße 31a, 8010 Graz, Austria; ^2^Center of Physiological Medicine, Institute of Physiological Chemistry, University of Graz, 8010 Graz, Austria; ^3^Faculty of Exercise and Sports Sciences, Center of Behavioural and Health Sciences, University of Tartu, 50090 Tartu, Estonia; ^4^Department of Pediatrics, Paracelsus Private Medical University of Salzburg, 5020 Salzburg, Austria; ^5^Practice for General Internal Medicine, Bruck a.d.M., 8600 Graze, Austria; ^6^Clinical Institute for Medical and Chemical Laboratory Diagnosis, Medical University of Graz, 8010 Graz, Austria

## Abstract

*Objective*. Risk phenotypes for cardiovascular disease (CVD) differ markedly between countries, like the reported high difference in CVD mortality in Austria and Estonia. Hitherto, the goal of this study was to find out risk profiles in body fat distribution yet present in childhood, paving the way for later clinical end points. *Methods*. he subcutaneous adipose tissue (SAT) distribution patterns in 553 Austrian (A) and Estonian (E) clinically healthy normal weight boys aged 11.1 (±0.8) years were analysed. We applied the patented optical device Lipometer which determines the individual subcutaneous adipose tissue topography (SAT-Top). *Results*. Total body fat did not differ significantly between E and A boys. A discriminant analysis using all Lipometer data, BMI, and the total body fat (TBF) yielded 84.6% of the boys correctly classified in Estonians and Austrians by 9 body sites. A factor analysis identified the SAT distribution of E as critically similar to male adult patients with coronary heart disease (CHD). *Conclusions*. We show in normal weight Estonian boys a highly significant decreased fat accumulation on the lower body site compared to age matched Austrian males. This SAT-Top phenotype may play an important role for the increased cardiovascular risk seen in the Estonian population.

## 1. Introduction

The risk for populational diseases differs markedly between geographic regions and countries, as, for example, the reported greater rate of cardiovascular mortality in Estonia compared to Austria. The latest age standardised ischemic heart disease (IHD) mortality data per 100 000 population are for Austria 80,68, while for Estonia 254,25 [1].

So far, the studies investigating cardiovascular disease (CVD) risk factors focused on adult obesity and associated metabolic disorders. However, for the assessment of populational health, it is of outmost interest to gain a better insight in the pediatric roots of CVD.

Although it is well established that obesity and the subcutaneous adipose tissue (SAT) phenotype are essentially involved in CVD risk, little information exists about risk phenotypes in prepubertal obese children.

Notably, the prevalence of childhood obesity increased 4-fold during the last 20 years [2], and this represents a strong risk factor for obesity of adulthood [3]. The SAT phenotype in children is of growing interest for later on CVD risk. An improved understanding of individual subcutaneous body fat distribution and body composition in children may essentially support the development of effective preventive strategies [4–7] for the metabolic syndrome and CVD [7–11].

Various methods to evaluate body composition in children exist. Due to the necessary technical equipment, some of these methods are very expensive besides being inappropriate for the use in field studies. The optical device Lipometer (EU Pat. no. 0516251) was developed to generate noninvasive, quick, accurate, and safe measurements of a monolayer of subcutaneous adipose tissue (SAT) at any given site of the human body. Technical features and validation results were based on computed tomography as reference system [12, 13]. The Lipometer allows the measurement of subcutaneous fat distribution and the determination of the subcutaneous adipose tissue topography (SAT-Top). Previous results using the Lipometer to determine the SAT-Top emphasize the importance of describing subcutaneous adipose tissue in adult obesity and metabolic syndrome as well as during childhood and adolescence [14–17]. 

Marked differences of mortality rates were seen between EU member states as analysed by the topology of male mortality [18]. This analysis yielded six groups of member states. Austria belongs to “type 1” with an almost average mortality, and Estonia is presented in “type 5” with high excess mortality from 20 to 64 years. Thus, we hypothesize herein the presence of high risk cardiovascular SAT-Top phenotype already present in Estonian children. 

This study analysed the subcutaneous body fat distribution in a sample of 553 clinically healthy male prepubertal boys aged around 11 years living in Austria or Estonia. 

## 2. Subjects and Methods

### 2.1. Subjects

The participants and their parents consented to the study after receiving a thorough explanation of the declaration of consent. The procedure chosen was in accordance with the Declaration of Helsinki and the local ethics committee recommendations. Height, weight, and SAT-Top were measured in 553 boys aged 11,1 (±0,8) years. Since there is a peak of fat mass in boys at the age of 11 years, we recruited individuals aged 10,0 to 12,0 years.

The participants were recruited in different schools and outpatient clinics from urban areas as well as in the local University Clinic for Pediatrics and Adolescent Medicine in Graz, Austria, and Tartu, Estonia. In Graz, boys of similar age were recruited from the STYrian Juvenile Obesity Study, which were designed to investigate early atherosclerosis and metabolic disorders in obese juveniles [19]. In Estland, boys were recruited from a running preventive study on obesity and exercise. None of the studied children suffered from endocrine or syndromal disorders or were on any medication. Height was measured using a stadiometer (SECA-220, Hamburg, Germany). Body mass was measured to the nearest 0.01 kg using calibrated electronic scales (Soehnle 7700, Murrhardt, Germany). 

Degree of overweight was calculated as body mass index (BMI). Height was measured to the nearest centimeter. Weight was measured in underwear to the nearest 0.1 kg using a calibrated balance scale. We used Box-Cox transformation to calculate BMI as a measure for degree of overweight due to the skewness of the BMI distribution [20]. Overweight was defined by a BMI above the 90th percentile for German children [21]. All children were also overweight according to the International Task Force for Childhood Obesity [22].

In addition to the reported group of Austrian and Estonian boys, we included the age development of 960 healthy controls aged from 9 (m09) to 80 years (m80) who were recruited from health and fitness checks in Austria and Austrian male adult patients with type 2 diabetes (T2DM), coronary heart disease (CHD), and persons with extremely thin fatty tissue layers (anorexia and body builders) (published in [15, 23] for visual comparison in a two-dimensional factor plot).

### 2.2. Measurement of Subcutaneous Adipose Tissue Topography (SAT-Top)

Measurements of SAT thickness were performed by means of a patented optical device (EU Pat. no. 0516251) on 15 anatomically well-defined body sites distributed from neck to calf on the right and left side of all children and then averaged for both body sides. The sensor head of the Lipometer, that is held perpendicular to the measurement site, consists of a light source of light-emitting diodes (*λ* = 660 nm, light intensity 3.000 mcd) and a photodetector that measures the corresponding light intensities that are back scattered in the SAT. Calibration and evaluation were done using computer tomography (CT) as the reference method. 

To obtain the Lipometer measurement values and the corresponding absolute values of CT for subcutaneous adipose tissue thickness, CT scans at defined levels (upper abdomen, lower abdomen, and extremities) were made by a third generation CT scanner (Somatom DR3, Somatom DHR, SIEMENS, Erlangen, Germany). These levels were marked, and Lipometer measurements were performed step by step around the body circumferences. Thus, a set of datapoints of both, Lipometer light patterns and corresponding absolute values of subcutaneous adipose tissue thickness from CT, ranging from 0.9 to 47.3 mm, was obtained. Final results were obtained by nonlinear regression (*r* = 0.9863), investigating different curve types such as polynomials from 2nd to 5th order, potential equations, and exponential equations. A detailed SAT profile of a subject is obtained by measuring the complete set of 15 specified body sites [12, 13].

### 2.3. Statistics

All statistical analyses were carried out using PASW Statistics 18.0 for Windows. Kolmogorov-Smirnov (KS) test was used to examine for normal distribution. If variables were not normally distributed, they were logarithmically transformed. Means were compared by a two-tailed unpaired sample *t*-test or by Mann-Whitney *U*-Test, depending on the distribution of the data. A value of *P* < 0.05 was considered statistically significant. 

Discriminant analysis undertakes the same task as multiple linear regression by predicting an outcome. Stepwise discriminant analysis is concerned with selecting the most important variables whilst retaining the highest discrimination power possible. Stepwise linear discriminant analysis was applied to study whether single SAT layers or the combination of SAT layers or TBF mass could enable correct classification between Estonian versus Austrian boys. 

**Figure 1:**
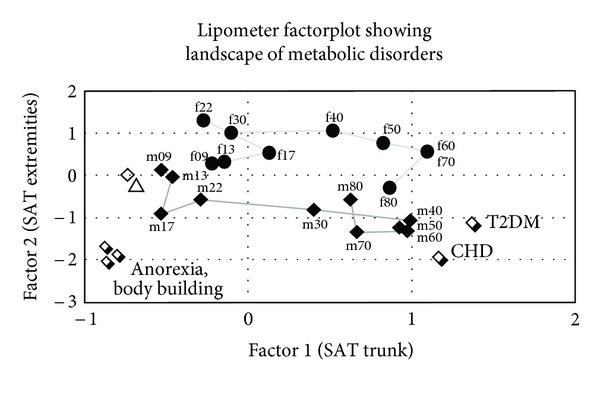
Factor analysis condenses the subcutaneous adipose tissue topography (SAT-Top) information at the trunk and the body sites at the extremities into a two-dimensional plot, where the position of each subject group is located (Austrian boys: rhombus; Estonian boys: triangle).

For visual comparison, the 15-dimensional SAT-Top information was condensed by factor analysis into a two-dimensional factor plot. The factor analysis shown in Figure 1 condenses the 15-dimensional SAT-Top information in a two-dimensional factor plot. Factor 1, the *x*-axis, represents the trunk body fat development, whereas Factor 2, the *y*-axis, represents the corresponding data of the extremities. 

## 3. Results

Table 1 shows basic anthropometric data of the experimental groups and the details of the Lipometer analysis encompassing Estonian (E) and Austrian (A) boys. 

A discriminant analysis using all 15 SAT-Top values, BMI, and the total body fat (TBF) in kg yields the following classification results: 84,6% of 553 boys are correctly classified in Estonians and Austrians selecting the body sites 1-neck, 2-triceps, 3-biceps, 5-front chest, 6-lateral chest, 7-upper abdomen, 10-hip, 12-lateral thigh, and 13-rear thigh for the discriminant function. 

The factor analysis shown in Figure 1 condenses the 15-dimensional SAT-Top information in a two-dimensional factor plot. Factor 1, the *x*-axis, represents the trunk body fat development, whereas Factor 2, the *y*-axis, represents the corresponding data of the extremities. The lower line in the figure shows the age development of healthy male controls aged from 9 (m09) to 80 years (m80). Nine-year-old boys have thin fat layers at the trunk. Near the age of 17 years, the young males typically have decreasing thicknesses of adipose tissue layers at the extremities. Between 17 and 40 years, the young males increase their fat layers on the trunk (m17, m22, m30, and m40). For comparison, the upper line shows the age-dependent SAT-Top development of females from 9 (f09) to 80 years (f80), respectively.

Austrians have thicker SAT layers at the extremities (higher values of Factor 2 see Figure 1), whereas Estonians show markedly stronger fat layers at their trunks. Notably, compared to Austrians, the position of obese Estonian boys is critically nearer to male adult patients with type 2 diabetes (T2DM) and coronary heart disease (CHD) in the factor plot (Figure 1).

## 4. Discussion

In this study, for the first time, a detailed SAT-Top analysis of Austrian and Estonian prepubertal boys is presented. The results show clearly a difference of the SAT distribution in the two populations. This observation might initiate more detailed future research to characterize deviations of SAT-Top underlying populational-based diseases. 

This prevalence remained high over the last 20 years. In 1990, it was estimated to be approximately three times higher than that of corresponding Swedish males in the same age group. Estonian females show approximately one-third of the mortality rate of males [24].

The importance of body fat measurements in children for the implementation of strategies to fight against the development of metabolic diseases is evident. Different approaches to ameliorate the health status of young individuals focused on body composition. Sex differences in the developing health risks from obesity are important to be included in terms of optimal risk management strategies. The incidence of obesity in boys is an upcoming growing problem in our society [25, 26]. 

The main pathologic sequels of metabolic diseases are undoubtedly driven by excess fat mass. However, the fat distribution is most probably more important than the total body fat mass [27–29]. Fat reference curves for children [9] show that boys have a relatively flat 50th centile varying between 15% and 18% body fat over the entire age range from 5 to 18 years with a peak at the age of 11 years. Variability increases up to the age of 11 years with a marked increase in positive skewness. Both skewness and variability fall after age of 11 years. Our data report directly on this critical age group of boys. Addo and Himes published new reference curves for triceps and subscapular skinfold thicknesses in US children and adolescents [30]. Especially in research settings, the SAT-Top determined by lipometry is an important valid anthropometric indicator of regional and total body fatness. The individual “landscape” of subcutaneous fat identified by the Lipometer is very specific for the adipose tissue phenotype caused by the interplay between genetic and environmental factors. An essential advantage of the Lipometer to other methods is the quick and noninvasive approach without radiation burden. 

The few studies which have examined so far ethnic differences in adiposity in children and adolescents used advanced body composition measurement techniques with potential side effects (radiation burden) such as dual-energy X-ray absorptiometry and densitometry [31]. 

Taken together, our results, achieved by a complex anthropometric analysis, identified for the first time in young male Estonians a SAT phenotype which is very similar to that of male adult patients with manifest type 2 diabetes (T2DM) and coronary heart disease (CHD). These observations may suggest a presence of a genetic risk predisposition for cardiovascular/metabolic abnormalities, which underlines the exceptional importance of early life style interventions for this population.

## Figures and Tables

**Table 1:** Descriptive characteristics (mean ± SD) and subcutaneous adipose tissue-topography (SAT-Top) measurements (the thickness of subcutaneous adipose tissue in mm at 15 specified body sites: from 1-neck to 15-calf) medians (range) of 553 Austrian and Estonian boys. Statistically significant differences (by Mann-Whitney *U*-test compared) in 15 defined body sites (from neck to calf) to Estonians. Results are expressed as mean ± SD analysed by Student's *t*-test (^+^ *P* < 0,05, ^++^ *P* < 0,01, and ^+++^ *P* < 0,001) or as median (25th–75th percentile) and Mann-Whitney *U*-Test (*P < 0,05, **P < 0,01, and ***P < 0,001) depending on the distribution of data.

	Austrians normal weight 280	Estonians normal weight 273	*P*
Age	11,2 (±0,9)	11,0 (±0,7)	
Height	146,7 (±8,2)	147,5 (±7,5)	
Weight	36,5 (±5,9)	37,3 (±5,5)	
1-Neck	2,8 (2,0–3,9)	1,5 (1,0–2,6)	0,000***
2-Triceps	7,3 (5,9–9,0)	6,4 (4,8–8,4)	0,000***
3-Biceps	3,1 (2,3–4,6)	2,7 (1,7–4,5)	0,000***
4-Upper back	2,6 (2,0–3,6)	2,2(1,3–3,6)	0,000***
5-Front chest	3,2 (2,3–5,6)	3,9 (2,2–6,6)	0,181 ns
6-Laternal chest	2,2 (1,8–3,2)	2,3 (1,3–4,9)	0,598 ns
7-Upper abdomen	2,8 (2,1–5,6)	2,5 (1,5–5,5)	0,006**
8-Lower abdomen	4,2 (2,5–8,1)	4,8 (2,5–8,6)	0,681 ns
9-Lower back	4,7 (3,6–7,4)	5,3 (3,2–8,2)	0,901 ns
10-Hip	3,7 (2,4–6,6)	4,7 (2,9–7,7)	0,020*
11-Front thigh	4,9 (3,8–6,4)	4,8 (3,1–6,2)	0,026*
12-Laternal thigh	6,1 (4,4–7,9)	5,1 (3,6–6,8)	0,000***
13-Rear thigh	4,3 (3,2–6,0)	4,5 (2,9–5,9)	0,537 ns
14-Inner thigh	6,0 (4,5–8,3)	5,7 (3,8–7,5)	0,002**
15-Calf	3,9 (2,9–5,1)	3,6 (2,5–4,8)	0,013*
BMI	16,9 (±1,5)	17,0 (±1,5)	0,152 ns
